# Combined Anterior Cruciate Ligament Reconstruction and Extra‐articular Tenodesis Using the Iliotibial Band With Femoral Press‐Fit Fixation: A Low‐Cost and Reproducible Technique

**DOI:** 10.1002/atn2.70187

**Published:** 2026-07-25

**Authors:** José Leonardo Rocha de Faria, Felipe Galvão Abreu, Vitor Barion de Pádua, Valdeci Manoel de Oliveira, Rafael Erthal de Paula, Yuri Di Cavalcanti, Naasson Trindade Cavanellas, José Paulo Gabbi Aramburú Filho, João Antonio Matheus Guimarães, Geraldo da Rocha Motta Filho, Phelippe Valente Maia

**Affiliations:** ^1^ Instituto Nacional de Traumatologia e Ortopedia, INTO Rio de Janeiro RJ Brazil; ^2^ Hospital e Maternidade Teresinha de Jesus, HTMJ Juiz de Fora Minas Gerais Brazil; ^3^ Universidade Federal de São Paulo, UNIFESP, Escola Paulista de Medicina São Paulo Brazil

## Abstract

The addition of a lateral extra‐articular reinforcement to anterior cruciate ligament reconstruction has been shown to enhance rotational stability and protect the intra‐articular graft. Although several techniques using the iliotibial band have been described, most rely on fixation devices that increase surgical cost and complexity. This Technical Note describes a low‐cost, reproducible technique for combined anterior cruciate ligament reconstruction and lateral extra‐articular tenodesis using the iliotibial band, with femoral press‐fit fixation for both grafts. This approach eliminates the need for fixation hardware, simplifies the procedure, and may facilitate its adoption in a wide range of surgical settings worldwide.

## Graphical abstract

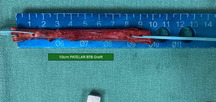

**VIDEO 1:** In an anterior cruciate ligament (ACL) rupture in a young, high‐demand patient presenting with clear instability—explosive pivot shift, grade 3 anterior drawer, and grade 3 Lachman—the proposed treatment is ACL reconstruction using the Chambat technique, associated with anterolateral reinforcement through extra‐articular tenodesis using the iliotibial band, with femoral press‐fit fixation. The next step is harvesting the bone–patellar tendon–bone graft. A longitudinal midline incision is made over the middle third of the patella and extended distally to the tibial tuberosity. The patellar tendon is marked for sectioning to obtain 10 mm width, 20 mm length, and 10 mm depth. With the graft on the back table, the patellar bone plug is shaped into a trapezoid measuring 15 mm wide proximally, 10 mm wide distally, 30 mm long, and 10 mm thick. After completing the diagnostic arthroscopy and creating the femoral and tibial tunnels, preparation of the iliotibial band graft is initiated. A 4‐cm oblique lateral incision is made, beginning 20 mm proximal to Gerdy's tubercle and oriented toward the lateral femoral epicondyle. After layer‐by‐layer dissection, the iliotibial band is visualized. Two parallel longitudinal incisions, spaced 1 cm apart, are made to create a strip. A transverse cut is then made 12 cm proximal to Gerdy's tubercle, preserving the distal insertion. This step is completed with a Krackow stitch applied to the free proximal end of the strip. The femoral tunnel is marked using the outside‐in technique, with the extra‐articular landmark located 5 mm proximal to the lateral femoral epicondyle—also the ideal location for the extra‐articular tenodesis tunnel. Drilling is performed from outside to inside, starting at the lateral femoral cortex and advancing into the joint. The lateral collateral ligament is identified, and the iliotibial band graft is passed beneath the ligament. After preparation of the femoral and tibial tunnels, a looped shuttle suture is passed through both tunnels and retrieved femorally. A transport knot is tied to allow passage of both the traction sutures from the iliotibial band graft and the traction sutures attached to the tibial plug of the ACL graft. The traction sutures are then pulled, advancing both grafts under arthroscopic visualization. Resistance is encountered when inserting the femoral plug, as its proximal portion is wider than its distal portion. An osseous impactor is then used to gently tap the graft into position. At the end of this process, both the extra‐articular tenodesis graft and the ACL graft are fixed in the femur through press‐fit compression. After completing the procedure, stability is reassessed using the same preoperative instability maneuvers, confirming a knee without signs of residual laxity. Video content can be viewed at https://doi.org/10.1002/atn2.70187.

Anterior cruciate ligament (ACL) reconstruction   (ACLR) is widely performed to restore knee stability after ligament injury. However, even with modern techniques, residual rotational instability and graft re‐rupture remain relevant concerns, particularly in young and active patients.^1^ The addition of a lateral extra‐articular procedure, whether an anterolateral ligament reconstruction or a lateral extra‐articular tenodesis (LET), has shown superior outcomes compared with isolated ACLR, reducing graft failure rates and residual pivot shift. As evidence accumulates, the combined ACLR and LET approach increasingly appears to represent the new gold standard in managing rotational instability.^1^, ^2^, ^3^, ^4^

Several methods for femoral graft fixation have been described, including interference screws, suspensory buttons, and transfemoral pins. Although effective, these techniques rely on fixation implants, and when an additional extra‐articular reconstruction is performed, the total cost of surgery may increase, posing challenges in resource‐limited settings. The press‐fit fixation technique offers an alternative, providing secure bone‐to‐bone fixation without the need for hardware, with proven biomechanical strength and favorable long‐term outcomes.^5^, ^6^, ^7^

The aim of this Technical Note is to describe a combined ACL and LET reconstruction using the iliotibial band (ITB) with femoral press‐fit fixation for both grafts, representing a simplified, low‐cost, and reproducible alternative for surgeons worldwide.

## SURGICAL TECHNIQUE

The surgical technique is presented in Video 1.

### Patient Setup

The patient is placed supine on the operating table in the standard arthroscopy position with a lateral post just proximal to the knee, at the level of the padded tourniquet, and a foot roll to prevent the hip from externally rotating and to maintain 90° of knee flexion. In this way, the knee can be moved freely through full range of motion. Appropriate landmarks are palpated and marked, including the joint line, Gerdy tubercle (GT) and lateral epicondyle.

### ACL Graft Harvesting

Traditionally, the graft is harvested through a single longitudinal midline incision extending from the inferior pole of the patella to the tibial tuberosity. After skin incision and exposure of the peritendon, two parallel incisions are made in the central third of the patellar tendon, approximately 1 cm apart, using a No. 15 blade. With the aid of an oscillating saw and a thin osteotome, the bone plugs are harvested in continuity with the tendon incisions.

**FIGURE 1:**
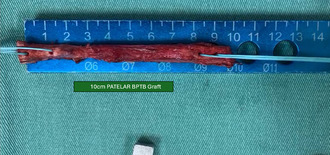
BPTB graft prepared with a tibial trapezoidal bone block, measuring approximately 2.5 to 3.0 cm in length, 1 cm in width, and 9 to 11 mm in thickness. The patellar bone block is rectangular, measuring approximately 2 cm in length, 1 cm in width, and 9 mm in thickness. (BPTB, bone–patellar tendon–bone.)

The patellar bone plug is rectangular, measuring approximately 2 cm in length, 1 cm in width, and 9 mm in thickness. The tibial bone plug is trapezoidal, measuring approximately 2.5 to 3.0 cm in length, 1 cm in width, and 9 to 11 mm in thickness (Figure 1). To minimize the risk of patellar fracture, it is recommended to drill small holes with a 2.0 mm Kirschner wire at both vertices of the patellar plug before final detachment with the osteotome.

After graft removal, two drill holes are created in the cortical surface of the tibial tuberosity (using a 2.0 mm Kirschner wire or a 3.2 mm drill bit) directed toward the cavity left by the tibial bone plug. Through these holes, two No. 5 Ethibond sutures (Ethicon, Somerville, NJ) are passed to allow for tibial fixation reinforcement later in the procedure. The tendon paratenon is then partially closed to minimize fluid leakage during arthroscopy.

### LET Graft Harvesting

**FIGURE 2:**
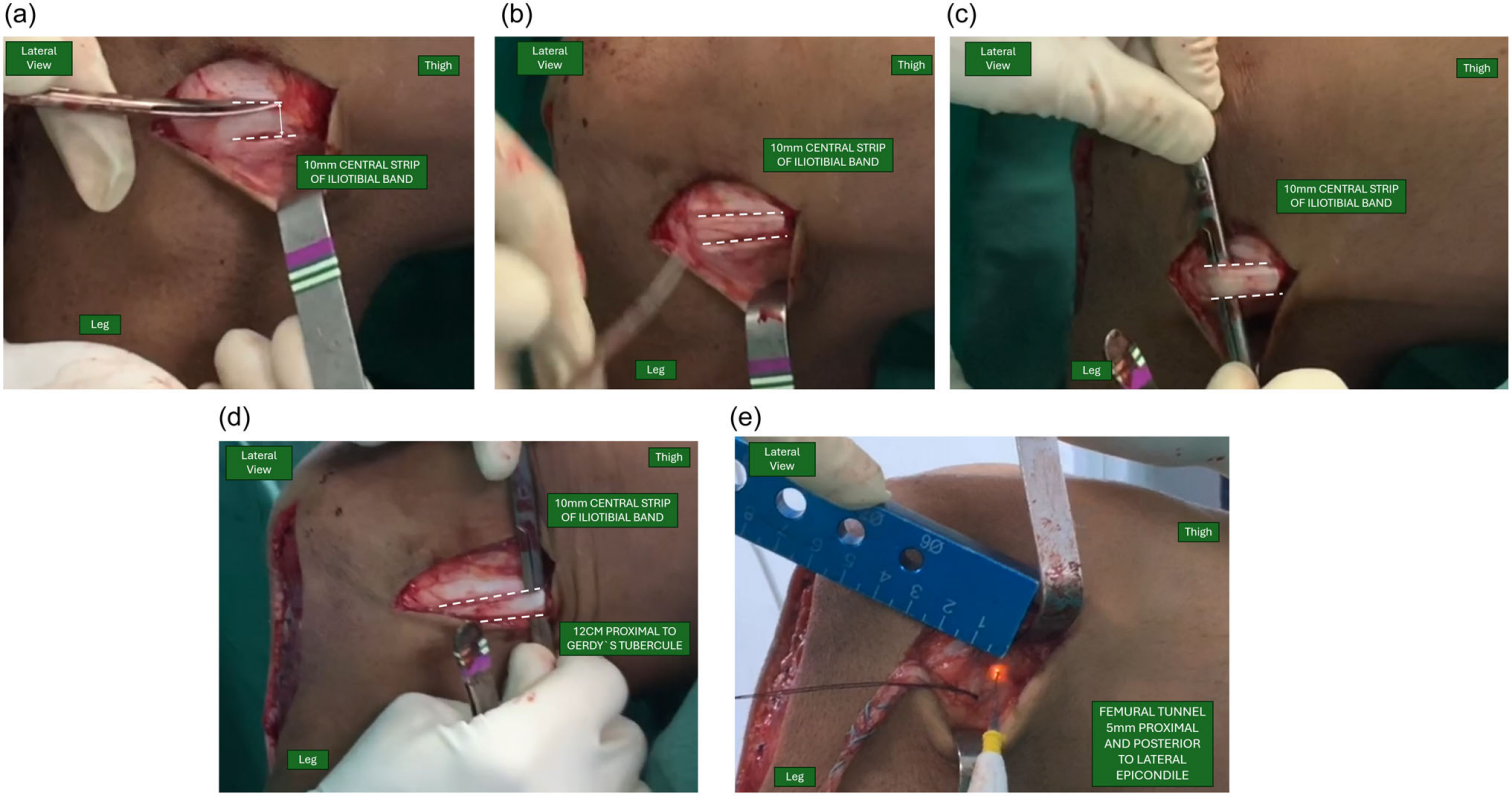
(a and b) A 10‐mm central strip of the ITB is harvested using Metzenbaum scissors. (c and d) The scissors are used to mobilize and release the central ITB graft, ensuring complete separation of any residual fibers. (e) A proximal incision is made to obtain a final ITB graft measuring approximately 12 to 13 cm in length. The femoral tunnel position for the ACL and the lateral extra‐articular tenodesis is pre‐marked—5 mm proximal and 5 mm posterior to the lateral epicondyle. Lateral view of the left knee. (ACL, anterior cruciate ligament; ITB, iliotibial band.)

A 4‐cm oblique lateral incision is made, starting approximately 20 mm proximal to GT and extending toward the lateral femoral epicondyle. The ITB is identified, and two longitudinal incisions are made parallel to its fibers, 1 cm apart, creating a strip of ITB (Figure 2). This strip is then transected transversely about 12 cm proximal to the GT, maintaining its distal insertion intact. The graft is carefully freed from the underlying fascia, and the proximal free end is tubularized using a strong nonabsorbable suture with Krackow stitches (Ethibond, Ethicon, Somerville, NJ, or FiberWire, Arthrex, Naples, FL).

After careful dissection, the lateral collateral ligament (LCL) is identified. Small incisions are made anterior and posterior to it, and a curved Mixter clamp is used to pass a shuttle suture beneath it. This suture will later be used to guide the graft from distal to proximal beneath the LCL.

### Arthroscopy and Tunnel Preparation

The arthroscopic portals are established through the previously incised skin. Standard anterolateral and anteromedial portals are created. After confirming a complete ACL tear, the remnants of the torn ligament are debrided, and both the femoral and tibial footprints are identified. With the knee flexed to 90°, the femoral origin of the ACL is debrided up to the most proximal edge of the lateral condyle.

**FIGURE 3:**
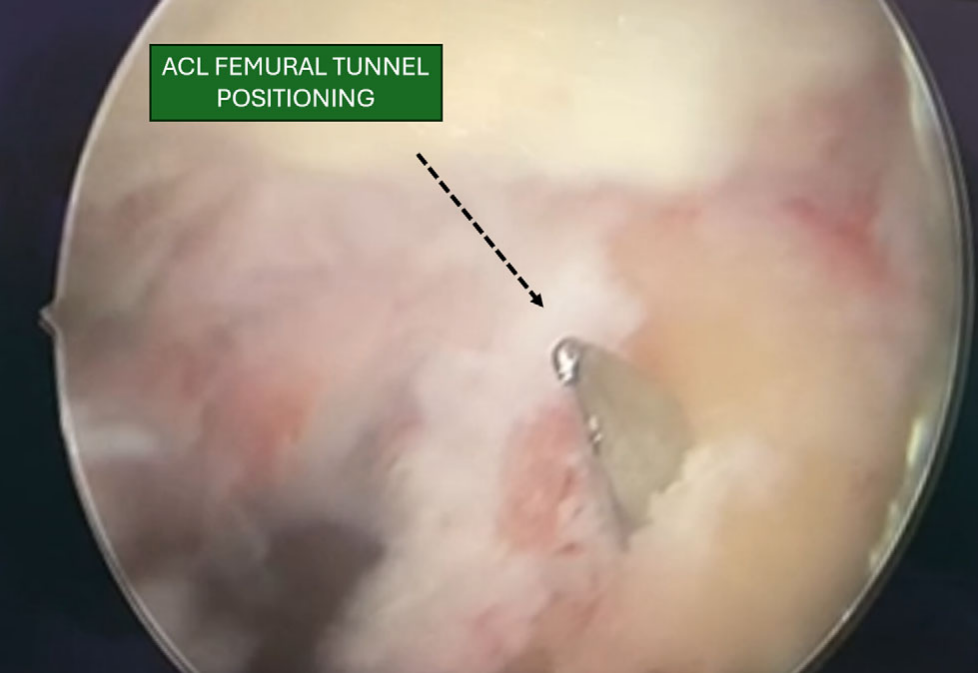
Femoral tunnel positioning using an outside‐in femoral guide, allowing anatomical articular and extra‐articular alignment. Arthroscopic view of the intercondylar region of the lateral femoral condyle, left knee. (ACL, anterior cruciate ligament.)

An outside‐in femoral guide (Síntegra Surgical—Pompéia—SP—Brazil) is introduced through the anteromedial portal and positioned at the femoral footprint of the ACL (Figure 3), engaging the most proximal region of the axial face of the lateral femoral condyle. The guide barrel, which is already aligned intra‐articularly, is positioned proximal and posterior to the lateral femoral condyle. A guidewire is inserted from outside to inside, and its position is confirmed arthroscopically. The femoral tunnel is then drilled from outside to inside, starting with a 6‐mm cannulated drill. Using a 6‐mm drill initially allows fine adjustments to tunnel positioning if needed. The tunnel is progressively enlarged up to 10 mm. Finally, an 11‐mm drill is used to perforate only the lateral cortex of the femur, taking care not to advance into the tunnel to preserve the bone bridge necessary for press‐fit fixation.

The tibial guide (Síntegra Surgical—Pompéia—SP—Brazil) is set at 55° and introduced through the anteromedial portal. The tip of the guide is positioned over the tibial footprint of the ACL, using the remaining native fibers, the medial tibial spine, and the anterior horn of the lateral meniscus as anatomical references. A guidewire is then inserted, and the tibial tunnel is created, starting with a 6‐mm drill and gradually enlarged up to 9 mm. A shaver blade is finally introduced into both tunnels to debride and remove any bone debris before graft passage. The remaining native fibers of tibial ACL footprint must be removed, to facilitate the passage of the bone–patellar tendon–bone graft.

### Graft Passage and Fixation

**FIGURE 4:**
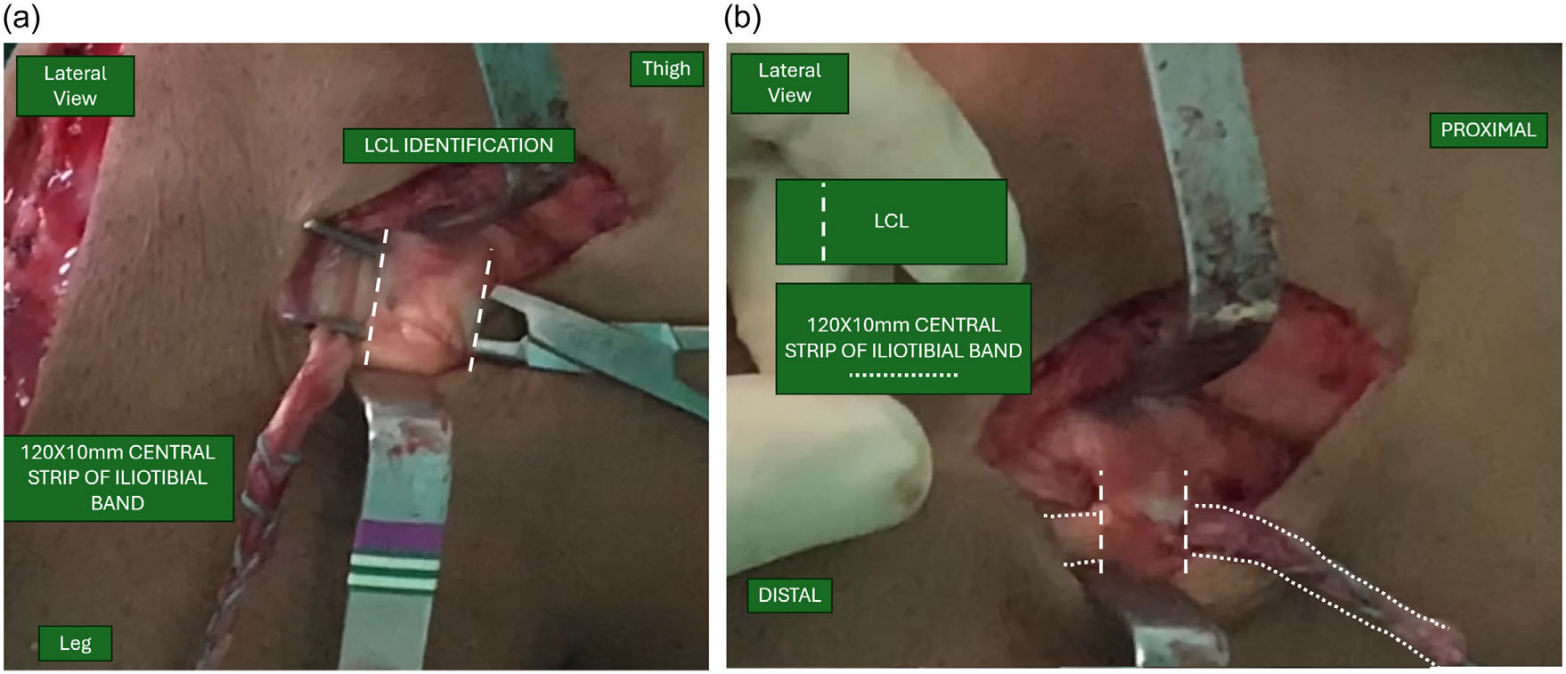
(a and b) Identification of the LCL, followed by passage of the ITB graft superficial to the LCL. Lateral view of the left knee. (ITB, iliotibial band; LCL, lateral collateral ligament.)

**FIGURE 5:**
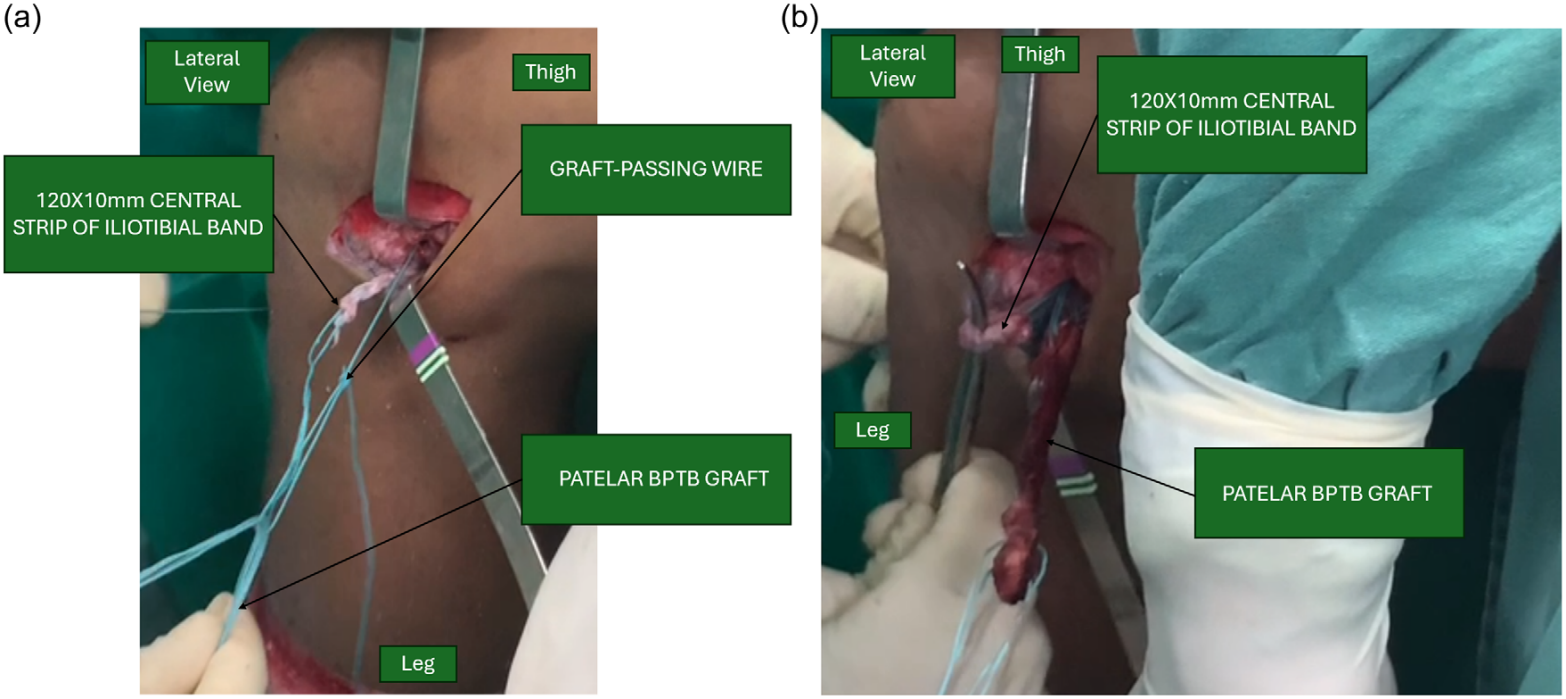
(a and b) The traction suture of the patellar BPTB graft (patellar bone block) and the traction suture of the ITB graft are both passed through the loop of the transport wire, allowing simultaneous advancement of both grafts from the femoral tunnel toward the tibial tunnel. (BPTB, bone–patellar tendon–bone; ITB, iliotibial band.)

Using the previously placed shuttle suture, the ITB graft is passed beneath the LCL from distal to proximal (Figure 4a and b). Afterward, a looped traction suture is introduced into the joint through the tibial tunnel and retrieved through the femoral tunnel using a grasper. This traction suture is then used to pull both the sutures attached to the ITB graft and those attached to the patellar bone plug of the ACL graft. The initial passage of the patellar plug through the femoral tunnel is facilitated with a straight Kelly clamp to guide its intra‐articular exit (Figure 5a).

**FIGURE 6:**
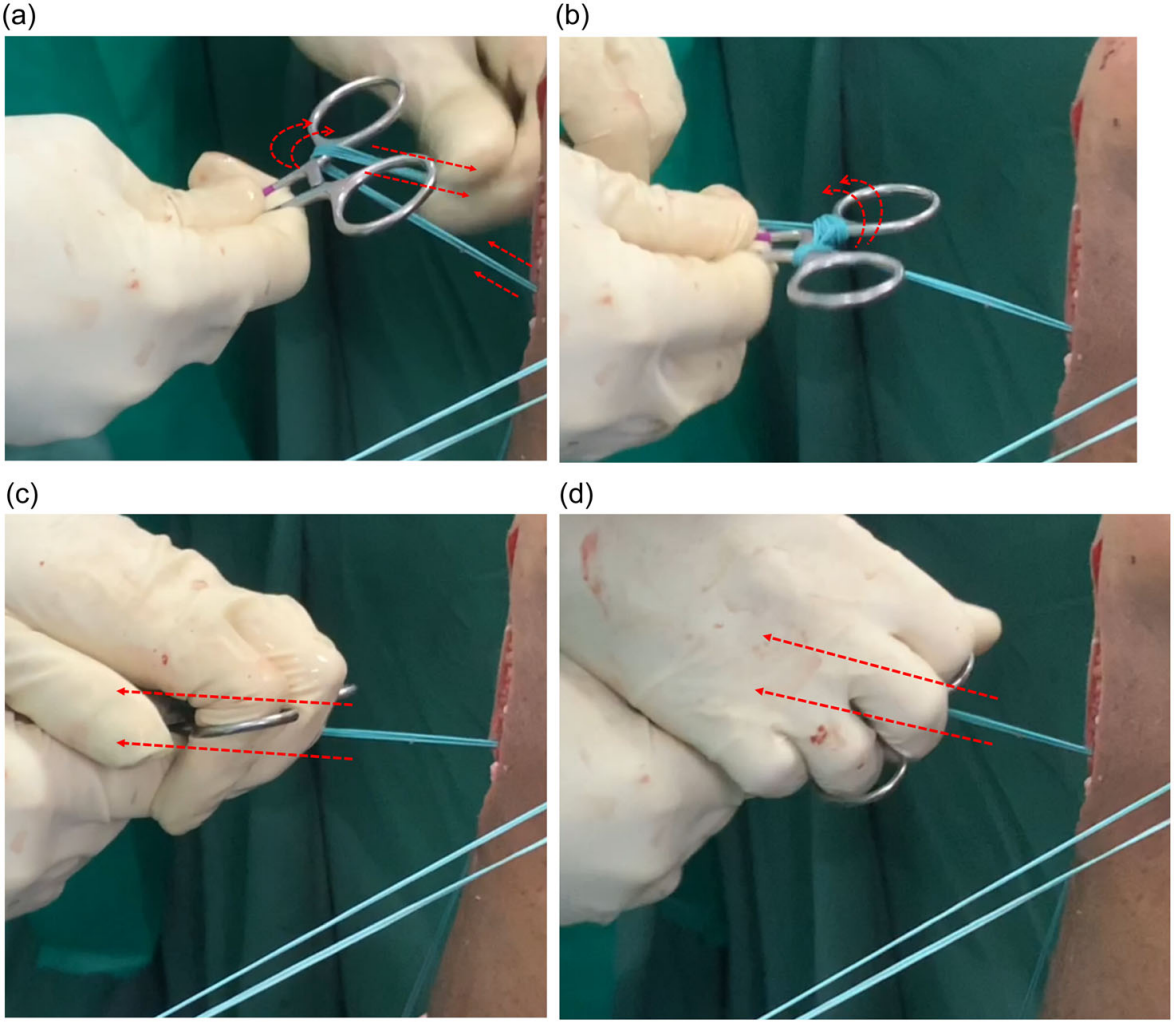
(a‐d) A Kelly clamp is used as an anatomical traction instrument. The traction suture is positioned around the metallic arches of the Kelly clamp to provide a stable handle for controlled traction of the transport sutures.

**FIGURE 7:**
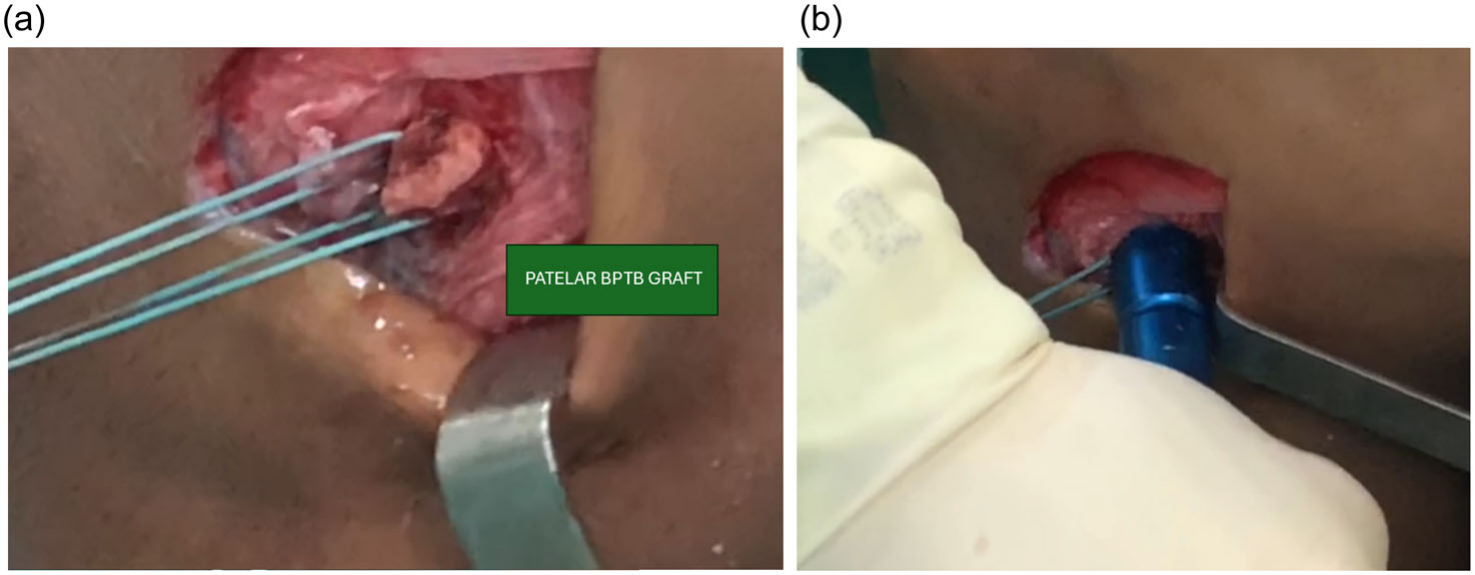
(a and b) With traction applied through the tibial tunnel, the trapezoidal tibial bone block of the BPTB graft engages the femoral tunnel aperture. The plug is then gently impacted using a bone impactor while traction is maintained on the graft. (BPTB, bone–patellar tendon–bone.)

With the knee maintained at 90° of flexion, the ACL graft is advanced through the tibial tunnel. As the patellar plug enters the tibial tunnel, resistance is typically encountered when the trapezoidal tibial plug engages the femoral tunnel (Figure 5b). At this point, a bone impactor is used to gently tap the tibial plug into the femoral tunnel while simultaneously pulling on the patellar plug and ITB traction sutures through the tibial tunnel (Figure 6a‐d and Figure 7a and b). Arthroscopic visualization confirms that the tendinous portion of the graft is entirely intra‐articular and that the patellar plug occupies the tibial tunnel. Once fully seated, both the LET and ACL grafts are fixed in the femur by the press‐fit compression mechanism.

**FIGURE 8:**
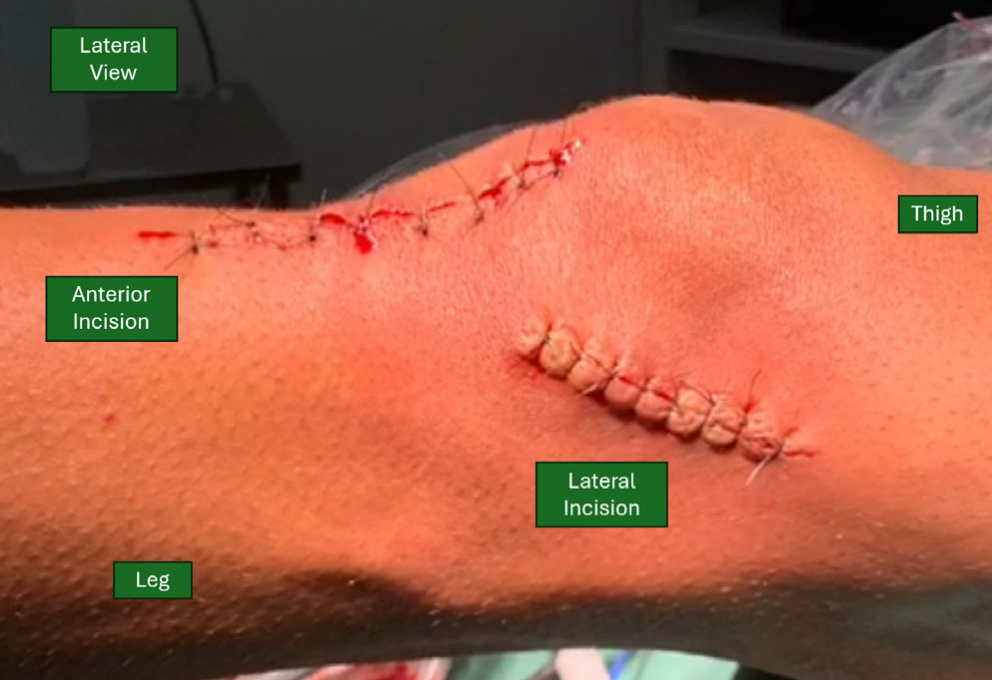
Final closure is performed with subcutaneous sutures followed by skin suturing in the standard fashion.

After graft passage, the knee is cycled through flexion and extension to accommodate and tension the graft. Final fixation is achieved by securing the patellar plug in the tibial tunnel with an 8 × 25 mm interference screw (Síntegra Surgical—Pompéia—SP—Brazil), with the knee positioned at 30° of flexion (Figure 8).

### Postoperative Protocol

Immediate full weight bearing without a brace and progressive range of motion exercises are allowed. Subsequent progression is milestone based.

## DISCUSSION

The described technique combines ACL reconstruction and LET using the ITB, both fixed with a femoral press‐fit method. This approach eliminates the need for additional fixation devices, thereby reducing surgical cost while maintaining strong biological fixation through full bone‐to‐bone contact within the tunnel. Press‐fit fixation has shown reliable biomechanical performance and long‐term success in ACL reconstruction.^8^, ^9^, ^10^

Compared with conventional techniques that require multiple implants, this method offers a simplified, low‐cost, and reproducible alternative, particularly suitable for surgeons in resource‐limited environments. The biological interface between graft and bone may also promote more physiological healing and reduce potential bone defects related to hardware use (Table 1).

**TABLE 1:** Pearls and Pitfalls

	Pearls	Pitfalls
Tunnel drilling	Gradual enlargement of the femoral tunnel with sequential drills allows minor adjustments in tunnel positioning before final sizing	It is crucial that the final drill does not advance deeply into the femoral tunnel and perforates only the lateral cortex, preserving the bone bridge necessary for proper press‐fit fixation
Passage of the LET beneath the LCL	Although the LCL is usually easily palpable, applying a force in the varus, or placing the knee in figure‐of‐4 position, can help	Placing Metzenbaum scissors deep to the LCL from anterior to posterior to bluntly dissect out a tract, and using a right‐angled clamp prevent the passage of the graft from being hampered by the soft tissues
	Suturing the graft to the LCL helps increase stability	
Graft fixation	When seating the bone plug into the femoral tunnel, gently tap it with a bone impactor to avoid excessive advancement or over‐insertion into the femur. After fixation, cycling the knee through repeated flexion‐extension movements helps the graft settle into its final position and minimizes the risk of future migration	Forceful impaction of the bone plug may drive it too deep into the femoral tunnel, compromising press‐fit fixation and reducing the bone‐to‐bone contact area essential for graft integration

LCL, lateral collateral ligament; LET, lateral extra‐articular tenodesis.

We advocate the use of this combined ACLR and LET technique in patients younger than 30 years, athletes, or those presenting with high‐grade pivot‐shift or undergoing revision surgery. The concept aligns with the current understanding that combined reconstructions enhance rotational control and reduce graft failure rates.^3^, ^11^

Several surgical techniques describe ITB tenodesis fixed at the classic Lemaire point, approximately 5 mm proximal and posterior to the lateral femoral epicondyle.^12^, ^13^, ^14^, ^15^ However, this location differs from the femoral insertion used for ACL graft fixation, thereby requiring the creation of two separate femoral tunnels. In contrast, performing fixation through a single shared femoral tunnel offers important advantages, including elimination of the risk of tunnel convergence, reduced surgical time by avoiding an additional drilling step, and lower overall procedural cost, regardless of whether fixation is achieved with an interference screw or a press‐fit technique.

A recent technique described a single‐tunnel approach for combined ACL reconstruction and LET, using hamstring autografts to assist ITB tenodesis.^16^ Manze et al.,^16^ in their description of the Over‐the‐Loop technique, detailed a graft‐handling strategy in which the hamstring graft is incorporated to augment lateral reinforcement while avoiding the creation of a separate femoral tunnel. This method highlights the growing interest in single‐tunnel configurations aimed at reducing tunnel convergence and optimizing graft positioning in combined procedures.

It is important to note that the technique described in this paper requires precise graft passage beneath the LCL and accurate identification of the femoral insertion point. Although technically demanding during the learning phase, these steps become reproducible after adequate anatomical familiarization.

A potential concern of femoral press‐fit fixation without supplemental hardware is the risk of graft fixation loss or early failure. Because fixation relies exclusively on precise bone‐to‐bone compression within the femoral tunnel, inadequate tunnel preparation, insufficient plug sizing, or excessive impaction may compromise stability. Furthermore, femoral bone plug fracture may occur during graft preparation or forceful impaction during insertion. These risks underscore the importance of meticulous graft preparation and controlled seating of the bone plug.

In our opinion, this procedure offers an advantageous balance between biomechanical stability, biological fixation, and surgical affordability. Advantages and disadvantages of the technique are summarized in Table 2.

**TABLE 2:** Advantages and Disadvantages

Advantages
Low‐cost procedure, requiring only 1 screw (tibial fixation)
Single anatomic tunnel for ACL/LET avoids tunnel convergence and preserve bone stock
Improved stability passing the ALL beneath the LCL
Femoral press‐fit fixation provides direct bone‐to‐bone healing
Simplified reproducibility, even in limited‐resource settings

Disadvantages
Lateral incision is required
Risk of femoral bone plug fracture during impaction
Potential risk of femoral fixation loss without supplemental hardware

ACL, anterior cruciate ligament; ALL, anterolateral ligament; LCL, lateral collateral ligament; LET, lateral extra‐articular tenodesis.

## DISCLOSURES

The authors (J.L.R.F., F.G.A., V.B.P., V.M.O., R.E.P., Y.D.C., N.T.C., J.P.G.A.F., J.A.M.G., G.R.M.F., P.V.M.) declare that they have no known competing financial interests or personal relationships that could have appeared to influence the work reported in this article.
